# ST-YOLO: A defect detection method for photovoltaic modules based on infrared thermal imaging and machine vision technology

**DOI:** 10.1371/journal.pone.0310742

**Published:** 2024-12-12

**Authors:** Hanfei Xie, Baoxi Yuan, Chengyu Hu, Yujie Gao, Feng Wang, Chunlan Wang, Yuqian Wang, Peng Chu

**Affiliations:** 1 School of Electronic Information, Xijing University, Xi ’an, Shaanxi, China; 2 Xi’an Key Laboratory of High Precision Industrial Intelligent Vision Measurement Technology, Xijing University, Xi’an, Shaanxi, China; 3 Graduate Office, Xijing University, Xi’an, Shaanxi, China; Purdue University, UNITED STATES OF AMERICA

## Abstract

Photovoltaic panels are the core components of photovoltaic power generation systems, and their quality directly affects power generation efficiency and circuit safety. To address the shortcomings of existing photovoltaic defect detection technologies, such as high labor costs, large workloads, high sensor failure rates, low reliability, high false alarm rates, high network demands, and slow detection speeds of traditional algorithms, we propose an algorithm named ST-YOLO specifically for photovoltaic module defect detection. This algorithm is based on YOLOv8s. First, it introduces the C2f-SCconv convolution module, which is based on SCconv convolution. This module reduces the computational burden of model parameters and improves detection speed through lightweight design. Additionally, the Triplet Attention mechanism is incorporated, significantly enhancing detection accuracy without substantially increasing model parameter computations. Experiments on a self-built photovoltaic array infrared defect image dataset show that ST-YOLO, compared to the baseline YOLOv8s, achieves a 15% reduction in model weight, a 2.9% improvement in Precision, and a 1.4% increase in mAP@0.5. Compared to YOLOv7-Tiny and YOLOv5s, ST-YOLO also demonstrates superior detection performance and advantages. This indicates that ST-YOLO has significant application value in photovoltaic defect detection.

## 1 Introduction

As the demand for sustainable energy continues to grow, the photovoltaic (PV) industry is experiencing remarkable development worldwide. Photovoltaic technology, with its unique environmental friendliness and sustainability, provides a reliable energy solution for modern society. Solar photovoltaic technology has become an important means of reducing dependence on fossil fuels, mitigating global climate change, and providing clean, green electricity. Relevant data shows that in 2022, power generation from solar and wind energy increased by 266 gigawatts, with a record high growth rate where solar energy accounted for 72% of the newly added power generation. However, with the rapid development of the PV industry, the widespread application of photovoltaics, while improving the efficiency of renewable energy utilization, also gradually reveals various challenges. The core of photovoltaic technology, the solar cell, is the key component that converts solar energy into electrical energy based on the photovoltaic effect produced by semiconductor materials under light. Despite the increasingly mature manufacturing process of crystalline silicon solar cells and modules, defects such as cold solder joints, broken grids, and micro-cracks may still occur during production. These defects can severely impact the performance and safety of PV panels. Adverse environmental conditions such as wind erosion, rain wash, and snow load can cause physical damage to PV panels, including surface cracks, delamination, or circuit interruptions. These defects significantly affect the energy conversion efficiency and power output performance of PV panels and increase potential circuit safety risks. Therefore, accurate and efficient defect detection technology for PV panels has become crucial for improving efficiency and ensuring product quality in the PV industry.

For defect detection in crystalline silicon photovoltaics, the industry currently widely uses technologies such as manual visual inspection, current-voltage (I-V) curve analysis, infrared thermal imaging, photoluminescence (PL) imaging detection, and electroluminescence (EL) imaging detection [1]. The manual visual inspection method is simple and quick, but it has limitations in detecting internal defects such as micro-cracks and cold solder joints. PL and EL imaging technologies generate detailed component images through laser irradiation and voltage application to reveal defects. Although these technologies have good detection performance, they are relatively complex and time-consuming to operate.

The visual inspection method is the most convenient and quick method; it only requires capturing the appearance of the component and imaging it on a display screen. This method is suitable for detecting defects such as spacing issues and foreign objects, but it is not suitable for detecting internal defects such as micro-cracks, cold solder joints, and cell issues. The I-V curve method determines whether there are defects in the component by observing the attenuation of I-V characteristics, but it cannot display the location and type of defects. It is mainly used to test the power and performance of the component.

The PL imaging detection method irradiates the cells or modules with a laser of a specific wavelength, causing ground-state electrons to transition to an excited state, forming electron-hole pairs. A photosensitive camera then receives the infrared light emitted at approximately 1150 nanometers as these electron-hole pairs recombine within a short period, thereby creating an image. The bright and dark areas in the imaging results directly reflect the concentration of minority carriers at different locations. Defect areas have a low concentration of minority carriers, appearing significantly darker in the imaging results and can be clearly identified. This method requires high-quality equipment, including a stable laser light source, but does not require forming a complete current circuit. It is mainly used for defect detection during cell production.

The EL imaging detection method is similar to the PL method. By applying a forward bias to the module, minority carriers are injected and recombine to emit light, which is received by a photosensitive camera to create an image. This method clearly shows the location and morphology of defects and has lower equipment requirements, making it widely used for defect detection in module production processes. Currently, in crystalline silicon photovoltaic production lines, EL imaging results are mainly judged manually, which is costly and inefficient. In recent years, researchers both domestically and internationally have begun to focus on the automatic detection of defects in EL images. Research methods are divided into two categories: traditional signal processing algorithms and artificial intelligence algorithms.

From the above analysis, it can be seen that the current-voltage (I-V) curve analysis method, PL imaging detection method, and EL imaging detection method are all used for defect detection in the production process of photovoltaic modules. However, a serious problem currently faced is the defect detection of photovoltaic modules during the operational phase of large-scale photovoltaic power plants.

The international community’s focus on renewable energy has driven the rapid growth of the photovoltaic (PV) industry. Green energy policies and the urgency of climate change issues have strongly supported the flourishing development of the PV industry. For instance, China’s northwest region, with its abundant solar resources, has established several large-scale PV power stations. For example, the “Super PV Station” project in Tongde County, Haixi Prefecture, Qinghai Province, has an installed capacity of several gigawatts, making it one of the largest PV power stations in the world. The Dunhuang PV Park in Gansu Province is renowned for its excellent sunlight conditions. The park hosts multiple PV power generation projects with a total installed capacity of hundreds of megawatts, making it a significant PV power production base in China. Zhongwei City in Ningxia, due to its ample sunlight and low land costs, has attracted several PV power generation projects. The construction of the Zhongwei PV base has effectively converted the region’s abundant solar resources, promoting local economic development and the aggregation of the new energy industry. The Hami region in Xinjiang is also an important PV power generation base in China. The PV power projects in this area have high generation efficiency due to the high number of sunlight hours and dry climate conditions. The Hami PV power stations not only supply power to the Xinjiang region but also provide clean energy to other provinces in China through the West-to-East Power Transmission Project. Correspondingly, the government has vigorously supported the development of PV enterprises, successively launching policies such as the “PV Leader Program.” In the latest national five-year plan, China has clearly outlined the development goals of the PV industry, emphasizing the crucial role of PV technology in the future energy structure, and proposed specific measures to support the development of the PV industry, such as accelerating the research and development and industrial application of PV technology. Additionally, the United States, Germany, and the European Union have each implemented region-specific support plans to promote the development of their domestic PV industries. In the United States, federal-level investment tax credits provide direct economic incentives for PV system installations, while various states’ feed-in tariffs and renewable energy credits further strengthen these incentives. Germany, through its innovative Renewable Energy Act, has established long-term fixed feed-in tariffs, ensuring the income of PV power producers and promoting the widespread application of PV technology. The EU has adopted an integrated multi-national approach, setting clear renewable energy targets that member states must adhere to through the Green Deal and Renewable Energy Directive, and providing necessary support measures to ensure these targets are met. These policies reflect the commitment of advanced economies to promoting innovation and sustainability in the PV industry. By offering policy incentives and economic support, they have laid a solid foundation for the prosperity and technological advancement of the PV industry. These policies and measures collectively form a comprehensive global support framework for the PV industry, aiming to drive technological progress, optimize energy structures, achieve cleaner and more efficient energy production, and promote sustainable economic development.

Photovoltaic (PV) modules are the most crucial components of PV power stations. During the operation of large-scale PV power stations, the safety issues of PV modules caused by the “hot spot effect” have been a constant concern for operators. The “hot spot effect” can cause point spots, stripe spots, and open circuits. If these issues are ignored or not detected in time, they may reduce the output power of the PV modules. If the heating temperature reaches a certain threshold, it can lead to localized burning of the PV modules, forming dark spots, solder joint melting, encapsulation material aging, and other permanent damages. These issues affect the output power and lifespan of the PV modules and can even pose safety hazards. Therefore, the detection of the “hot spot effect” in PV modules during the operation of large-scale PV power stations is extremely important.

As previously explained, the current-voltage (I-V) curve analysis method, infrared thermal imaging method, PL imaging detection method, and EL imaging detection method are all used for defect detection in the production process of PV modules. The technology that can be applied for detecting the “hot spot effect” in PV modules during the operation of large-scale PV power stations is the infrared thermal imaging method. Infrared thermal imaging is a real-time, non-destructive defect imaging technology [2]. It achieves precise localization of defects by capturing the thermal radiation generated by local overheating due to current consumption at defect sites. For example, in our self-constructed PV module infrared image defect dataset, there are several types of defects such as DB (dot spots), TB (strip spots), and DL (disconnections). Dot spots are usually caused by local short circuits or material defects. These small areas may overheat due to concentrated current flow, forming a small spot with a higher temperature. Strip spots may be caused by uneven current distribution within the module or damage to local conductive paths, leading to concentrated current in a strip-shaped area and resulting in overheating in that region. When a disconnection occurs inside a PV module, current cannot pass through the affected area, leading to a local power generation halt. These defects can cause changes in the electrical characteristics within the PV module, resulting in abnormal temperature distributions. Therefore, infrared thermography has become an effective tool for detecting and diagnosing defects in PV modules, as it can intuitively reflect the electrical defects inside the modules. Additionally, this technology can provide real-time image output, speeding up the detection process and improving work efficiency. Its value is particularly prominent in application scenarios requiring rapid assessment of a large number of PV panels. Infrared thermal imaging can intuitively reveal hotspots and thermal effects caused by defects, helping to identify potential electrical faults early and prevent further damage caused by thermal effects. Another advantage of the infrared thermal imaging method is its ease of integration and automation. This paper employs a method where unmanned aerial vehicles (UAVs) carry infrared thermal imaging equipment, combined with deep learning technology, to achieve large-scale, efficient automated detection.

## 2 Related work

Photovoltaic panel defect detection mainly focuses on electroluminescence (EL) imaging technology, photoluminescence (PL) imaging technology, and infrared thermal imaging technology. The following introduces their related work.

Based on electroluminescence (EL) imaging technology, Hassan Eesaar et al. proposed the SEiPV-Net model. This model uses an encoder-decoder-based network architecture that can autonomously segment 24 defects and features in electroluminescence images of solar PV modules. The superiority of this framework is demonstrated through comparisons and visual evaluations using state-of-the-art (SOTA) technologies [3]. Aidong Chen and colleagues proposed an improved anomaly detection method based on Faster R-CNN for surface defects of PV cells. This method integrates lightweight channel and spatial convolutional attention modules, which can more effectively analyze crack defects in complex scenes [4]. Harsh Rajesh Parikh et al. extracted statistical parameters from the histograms of EL images and used them as feature descriptors. Then, machine learning algorithms were used to train these descriptors. Experiments showed that the proposed method could autonomously detect cracks and finger faults [5]. Fatma Mazen Ali Mazen et al. proposed an automatic PV defect detection system for EL images by improving YOLOv5. The global attention module (GAM) was incorporated into the traditional YOLOv5s model to better represent objects, adaptive feature space fusion (ASFF) was added to the original structure of YOLOv5 for feature fusion, and distance intersection over union non-maximum suppression (DIoU-NMS) was aggregated to generate more accurate bounding boxes. Experimental results showed that test-time augmentation (TTA) significantly improved the mAP@0.5% to 77.7%, surpassing the YOLOv8 model under the same conditions [6]. Xin Chen et al. proposed a fast semantic segmentation method to automatically segment cracks and extract crack features in EL images. By fine-tuning the UNet neural network model using a pre-trained VGG16 as the encoder, they achieved an average F1 score of 0.875 and an intersection over union score of 0.782 on the test set [7]. To address power loss issues in solar panels during field operations due to faults or performance degradation, Stefan Bordihn et al. combined principal component analysis (PCA) and k-nearest neighbors (kNN) classifiers to achieve automatic detection of potential induced degradation (PID) and light and elevated temperature-induced degradation (LeTID) faults in solar panels, with prediction performance reaching expert levels [8]. Zhou Ying et al. proposed a defect detection algorithm based on YOLOv8, named YOLOv8-EL. They used GauGAN for data augmentation, embedded a context aggregation module (CAM) between the backbone network and the feature fusion network, and constructed a multi-attention detection head (MADH) to replace the decoupling head, extracting key information at the spatial and channel levels and reducing feature confusion. Experimental results showed that the model achieved an average precision of 89.90% on the augmented PV cell EL dataset, with a model parameter size of 13.13M. Subsequent generalization experiments on the PASCAL VOC dataset demonstrated the generalization performance of the improved algorithm [9]. Ai Shangmei et al. proposed a novel PV module defect detection method based on the SSD algorithm, integrating a super-resolution network and dual-pooling feature extraction. By using a generative adversarial network to expand the dataset and improving the VGG19_MP network to enhance texture feature analysis, higher detection accuracy and efficiency were achieved [10].

Based on photoluminescence (PL) imaging technology, Bernd Doll et al. combined high resolution and high throughput to propose a PL imaging method suitable for outdoor full-size module imaging. This method does not require electrical contact and provides image resolution comparable to that of electroluminescence (EL) images [11]. Marija Vuković et al. proposed a photoluminescence imaging method that does not use lock-in techniques to filter sunlight. This method extends its application to diffuse irradiance conditions, showing that it provides valuable information about the module even when image acquisition is performed under diffuse global irradiance as low as 40 W^^m-2^^. The PL images obtained at low irradiance are comparable to the EL images obtained at 10% of the short-circuit current [12].

Based on infrared thermal imaging, Al Reem Majid Ali Al Risi et al. proposed a non-invasive imaging system designed to install a thermal camera on an unmanned aerial vehicle (UAV) for thermal imaging of solar panels. Subsequently, various image processing techniques are applied to the captured images to extract relevant geometric and statistical features. Finally, machine learning classifier algorithms are used to analyze this processed data to identify areas with defects [13]. -Additionally, significant progress has been made in the field of image processing with respect to image segmentation methods for thermal imaging technology. Yawen Lu and colleagues enhanced the application accuracy of infrared thermography by matching thermal images with visible light images through thermal feature exploration. This research laid the groundwork for subsequent image processing technologies based on thermal imaging [14]. Jitesh Jain and his team introduced the general Transformer model, OneFormer, which performed excellently across various image segmentation tasks, showcasing the great potential of a single model in handling complex segmentation tasks [15]. Furthermore, Yawen Lu and colleagues developed a label-efficient video object segmentation method using motion cues, which reduces dependence on large amounts of labeled data and is suitable for large-scale applications in dynamic scenes [16]. Xiangtai Li and other researchers further explored the effectiveness of a single model in multiple image segmentation tasks, offering new ideas for simplifying multi-task processing workflows [17]. Sherozbek Jumaboev et al. utilized three segmentation models (FPN, U-Net, and DeepLabV3+) to propose a deep learning-based method for PV defect detection, using UAV thermal imaging to identify defective solar panels in large farms. This study demonstrates that deep learning-based image segmentation algorithms outperform traditional methods and can effectively address thermal imaging defect detection of solar panels [18].

In summary, while electroluminescence (EL) and photoluminescence (PL) imaging technologies have demonstrated their effectiveness in detecting PV panel defects, thermal imaging technology has a broader range of applications. Firstly, infrared thermal imaging can directly detect thermal anomalies in PV modules, making it extremely effective for identifying issues caused by efficiency reduction or damage. Secondly, this technology is suitable for a wide range of environmental conditions, especially outdoors, where it can perform effective detection under natural light—something that is difficult to achieve with PL and EL methods. This is because PL imaging requires irradiating the cells or modules with a laser of a specific wavelength, and EL imaging requires applying a forward bias to the module to inject carriers. Additionally, the non-contact nature of infrared thermal imaging makes it more convenient and safer for detecting large PV power stations. Therefore, infrared thermal imaging demonstrates broader applicability and higher efficiency in practical applications, making it especially suitable for the detection and maintenance of large-scale outdoor PV power stations. Consequently, in the detection of large-scale outdoor PV power stations, thermal imaging technology is preferred due to its efficiency, non-contact nature, and practicality.

**Fig 1:**
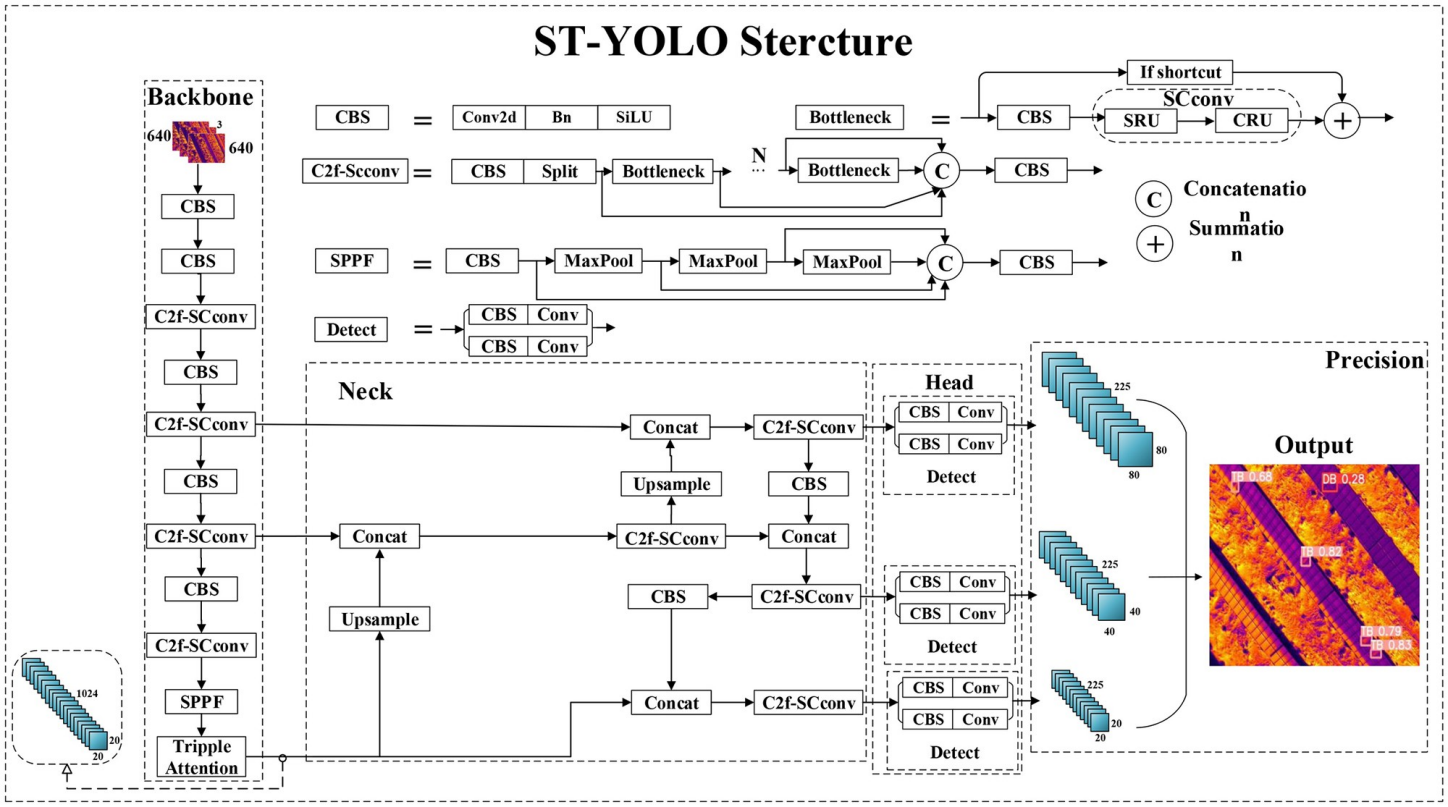
ST-YOLO model structure diagram.

Based on the experiences of the aforementioned researchers and the summary of existing photovoltaic module defect detection methods, this paper proposes ST-YOLO, specifically designed for photovoltaic module defect detection, using an infrared defect image dataset of photovoltaic arrays. Compared to traditional methods, ST-YOLO not only improves detection accuracy and speed but also significantly reduces computational resource consumption, making it more advantageous in practical applications. The framework of ST-YOLO is shown in Fig 1. The innovations of this algorithm are in the following aspects:

In view of the fact that we did not find publicly available datasets related to infrared image defect detection of PV modules in the relevant literature, we used UAV technology to collect infrared images of PV modules. The construction of this self-built dataset aims to ensure the diversity and representativeness of the data to support in-depth research on infrared image defect detection of PV modules.

The adoption of a deep learning-based infrared image detection algorithm for PV modules significantly reduces the cost of manual inspection and greatly improves the accuracy and efficiency of PV defect detection.

To achieve a high-precision algorithm and ensure its real-time performance, we chose the widely used YOLOv8s as the basic framework. Furthermore, to optimize the lightweight characteristics of the model, facilitate deployment, and improve computational efficiency, this paper introduces the SCconv convolution module.

To enhance the model’s ability to identify and locate PV panel defects, we integrated the Triplet Attention mechanism into the algorithm’s backbone. This step effectively improved the detection accuracy of the model.

The remainder of this paper is structured as follows: Chapter 3 provides a detailed introduction to our method and the related module structures. Chapter 4 presents our PV array infrared defect image dataset and the experimental environment, followed by various comparative and ablation experiments, and analyzes the results. Finally, Chapter 5 offers the conclusion.

The method proposed in this paper not only achieves significant improvements in the accuracy and efficiency of PV defect detection but also maintains the real-time performance of the algorithm. This is of great significance for promoting the development of the PV industry and enhancing the operational efficiency of PV systems.

## 3 Dataset and method

### 3.1 Dataset

The photovoltaic array infrared defect image dataset used in this paper was collected from a photovoltaic power station in the northwest region. The data collection process involves four main parts: photovoltaic arrays in natural scenes, a UAV-mounted data collection platform, carefully designed flight routes, and the collected photovoltaic array images. The collection process primarily relies on the DJI Matrice 300 RTK UAV flight platform and the DJI Zenmuse H20T gimbal camera. The Matrice 300 RTK UAV has a single flight time of 55 minutes and a flight payload of 2.7 kilograms, ensuring the efficiency and reliability of the photovoltaic array infrared defect image collection. The H20T gimbal camera integrates multiple sensors, including a 20-megapixel zoom camera, a 12-megapixel wide-angle camera, a laser rangefinder with a range of 1200 meters, and a thermal imaging camera with a resolution of 640 × 512 pixels, fully meeting the needs of photovoltaic array infrared image collection.

**Fig 2:**
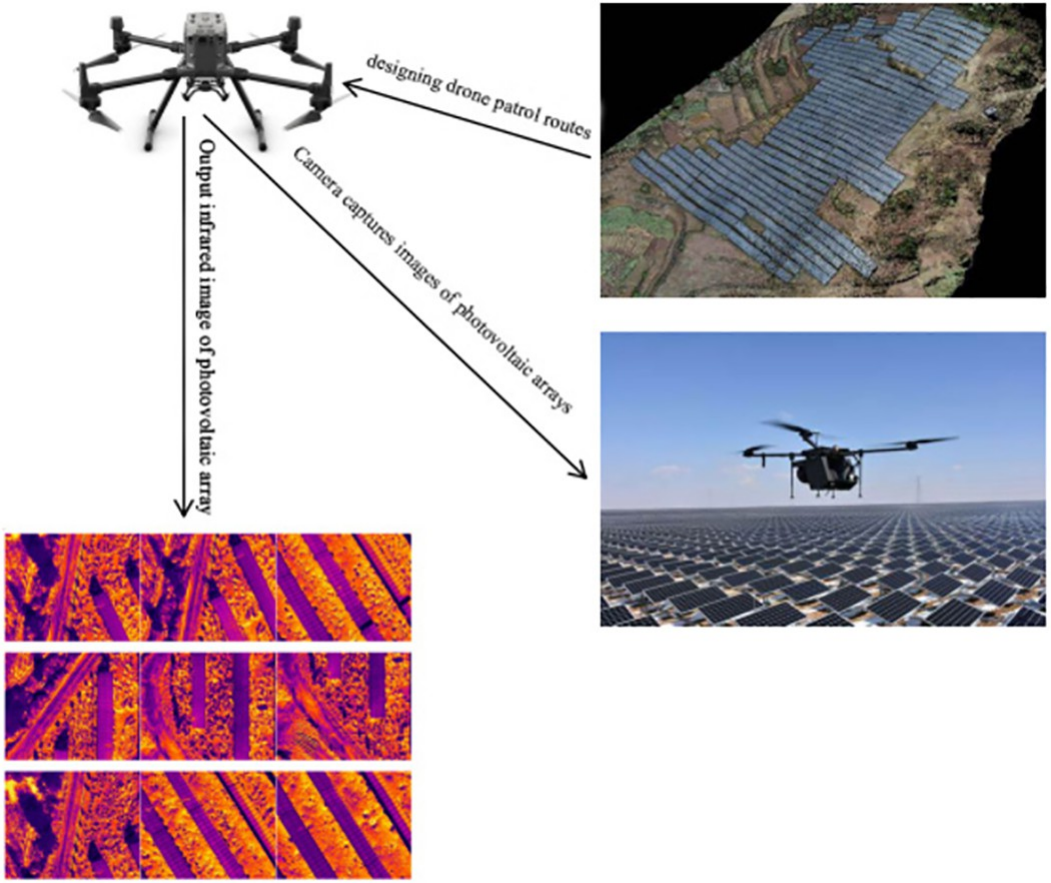
UAV image collection process diagram.

Based on the actual layout of the photovoltaic array, this study set specific flight routes for the UAV and set the shooting height to 100 meters above the ground to obtain images from the optimal perspective. Through this efficient data collection method, we gathered a large number of high-definition images of photovoltaic panels. These captured images were processed to form the infrared defect image dataset of photovoltaic arrays used in this research. Fig 2 illustrates the specific UAV image collection process.

Based on the above collection process, we successfully obtained tens of thousands of clear infrared images of PV modules. When addressing three obvious defect features in PV modules—point spots (DB), stripe spots (TB), and open circuits (DL)—we selected 1,692 representative infrared images of PV panels and had experts manually annotate them, ultimately creating the PV array infrared defect image dataset. This dataset is divided into 1,524 images for training, 168 images for validation, and the remaining unannotated images for testing. Each image has a size of 640 × 512, ensuring that each image contains at least one defect feature.

**Fig 3:**
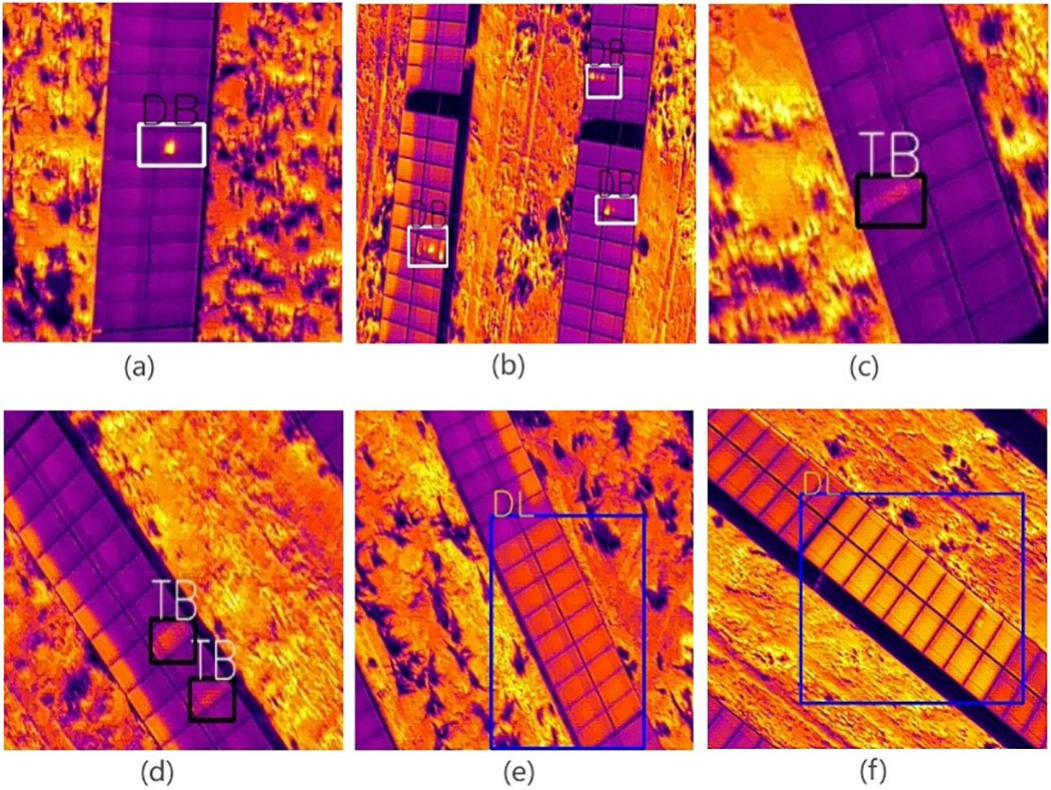
An example of a labeled dataset with DB, TB, and DL tags representing three types of defects: Dot defect, stripe defect, and disconnection.

Regarding specific defect types, point spots (DB) are usually formed due to localized overheating caused by low efficiency of the silicon wafer or surface contamination. Stripe spots (TB) may result from broken or detached solder ribbons leading to poor current flow. Open circuit defects (DL) are caused by a break in some part of the circuit, preventing smooth current flow. Fig 3 shows different features of the dataset.

### 3.2 ST-YOLO

In recent years, deep learning, as an important branch of machine learning, has made significant progress, particularly in convolutional neural networks. These networks, with their powerful feature extraction and nonlinear representation capabilities, have gradually been widely applied in various object detection scenarios. Deep learning object detection algorithms can generally be divided into single-stage and two-stage categories. In practical industrial applications, due to the importance of real-time performance, single-stage object detection algorithms have garnered significant attention and application [19], such as YOLO [20] and SSD [21], and are widely used in scenarios such as safety helmet detection, face recognition, and metal surface defect detection. Yuan Dai et al. proposed the YOLO-Former model, which improves global information capture ability and feature representation capability while reducing computational complexity by introducing the Vision Transformer (ViT) and Convolutional Block Attention Module (CBAM). Experiments show that YOLO-Former outperforms existing methods on the Foreign Object Debris Detection (FODD) and PASCAL VOC datasets while maintaining real-time processing speed [22]. Qinggang Wu et al. proposed an improved YOLOv5s method for object recognition in high-resolution remote sensing images. This method enhances the detection performance of dense targets in remote sensing images by pruning unnecessary residual modules and introducing a residual coordinate attention module, differential evolution algorithm, AW-IoU loss function, and SCYLLA soft non-maximum suppression [23].

Xiaohong Qian et al. proposed a novel industrial defect detection model “LFF-YOLO,” which employs the ShuffleNetv2 feature extraction network and a lightweight feature pyramid network, effectively improving detection speed and accuracy. It achieved a mAP of 79.23% on the NEU-DET dataset, 93.31% on the Peking University PCB defect dataset, and 59.78% on the GC10-DET dataset [24]. Tangbo Bai et al. proposed the RSG-YOLO model to address the specific needs of crack detection in ballastless track slabs. By adopting a dual fusion feature pyramid structure and GAM attention mechanism, the model significantly improved the accuracy and recall rate of crack detection in ballastless track slabs, specifically improving crack detection accuracy by 4.34% and mAP@0.5 by 3.08%, effectively solving the issues of missed and false detections [25].

This paper proposes ST-YOLO, a method specifically designed for photovoltaic module defect detection, based on a photovoltaic module infrared image dataset. To achieve a high-precision algorithm while ensuring real-time performance, we selected the widely-used YOLOv8s [26] model as the foundational framework. Furthermore, to optimize the model’s lightweight characteristics, facilitate deployment, and improve computational efficiency, this paper introduces the SCconv convolution module.

The SCconv module, proposed by Li et al. [27], is a novel spatial and channel reconstruction module. It is a plug-and-play architectural unit that can directly replace standard convolutions in various convolutional neural networks. This module consists of two components: the Spatial Reconstruction Unit (SRU) and the Channel Reconstruction Unit (CRU).

**Fig 4:**
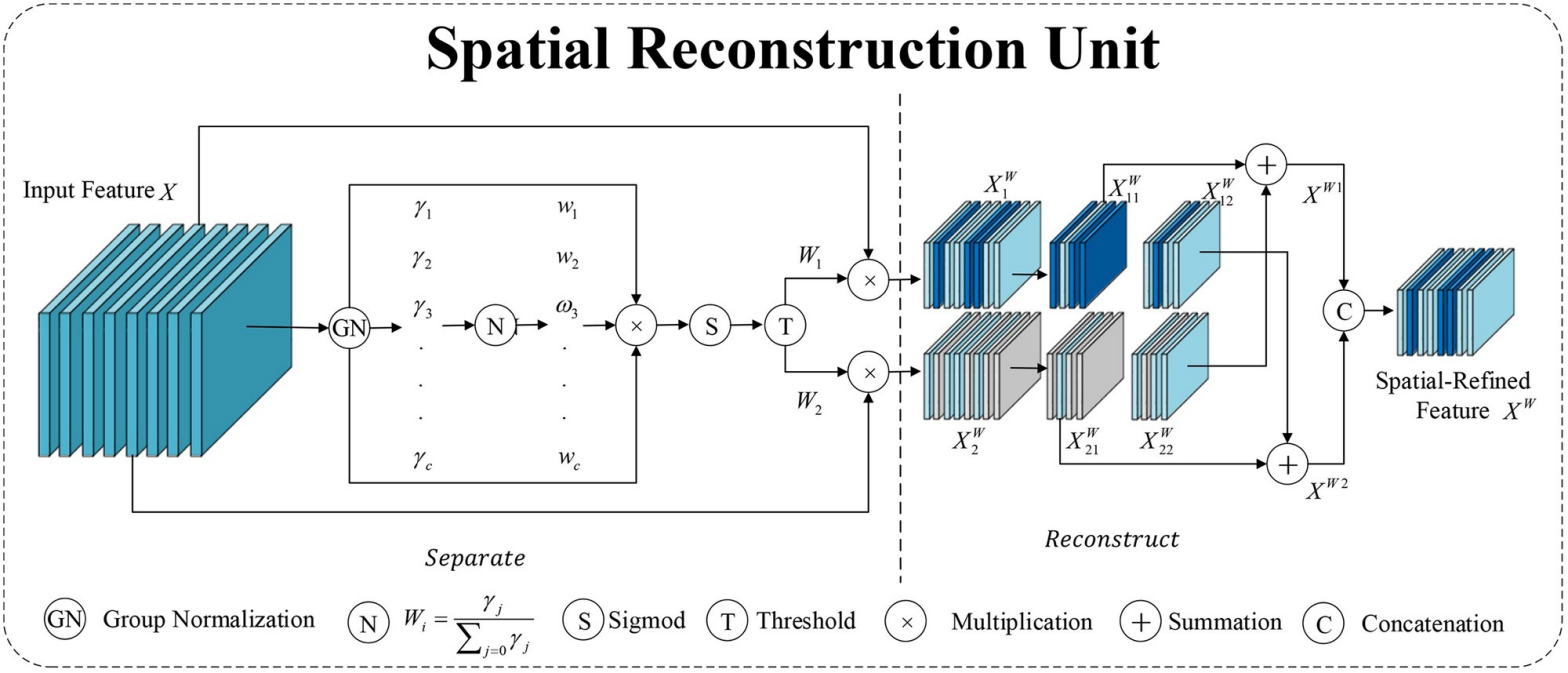
SRU component structure diagram.

Fig 4 shows the structure diagram of the Spatial Reconstruction Unit (SRU) component. The SRU suppresses spatial redundancy through a reconstruct-separate method. First, the input features undergo group normalization (GN), where the scaling factors in the GN layer evaluate the information content of different feature maps. Then, a mapping transformation is performed, and the input features are divided into two parts: one with rich spatial information and the other with less. These two parts are then cross-reconstructed, and finally, the two newly reconstructed feature spaces are concatenated to obtain the final output. The SRU suppresses spatial redundancy and improves the utilization of image space.

**Fig 5:**
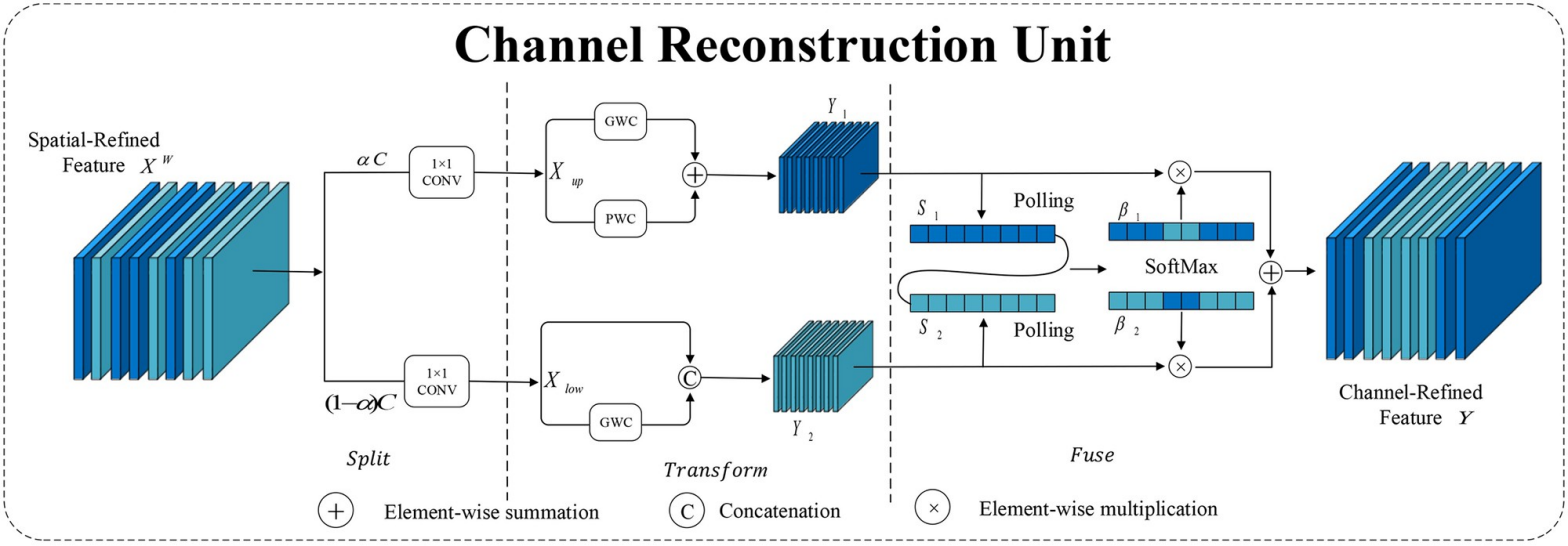
CRU component structure diagram.

Fig 5 shows the structure diagram of the Channel Reconstruction Unit (CRU) component. The CRU reduces channel redundancy through a split-transform-fuse method. The input spatial features *X*_*up*_ are divided into upper feature space and lower feature space *X*_*low*_. The upper feature space contains richer features and information, while the lower feature space contains less information. Then, the upper feature space *X*_*up*_ is processed separately using group convolution (GWC) and pointwise group convolution (PWC), followed by addition. The lower feature space *X*_*low*_ undergoes PWC processing and is concatenated with the lower feature space *X*_*low*_, resulting in feature *Y*_1_ with rich information and feature *Y*_2_ with less information. Finally, *Y*_1_ and *Y*_2_ undergo pooling operations to obtain *S*_1_ and *S*_2_. *S*_1_ and *S*_2_ are combined and processed by Softmax to obtain the feature importance vectors *β*_1_ and *β*_2_, which are then element-wise multiplied with the corresponding *Y*_1_ and *Y*_2_ and concatenated to obtain the channel refinement feature *Y*. The CRU further reduces the redundancy of the features generated by the SRU.

The SCconv module effectively limits feature redundancy through the SRU and CRU structures. Therefore, the SCconv module can reduce the computational load of model parameters while also enhancing the model’s performance.

**Fig 6:**
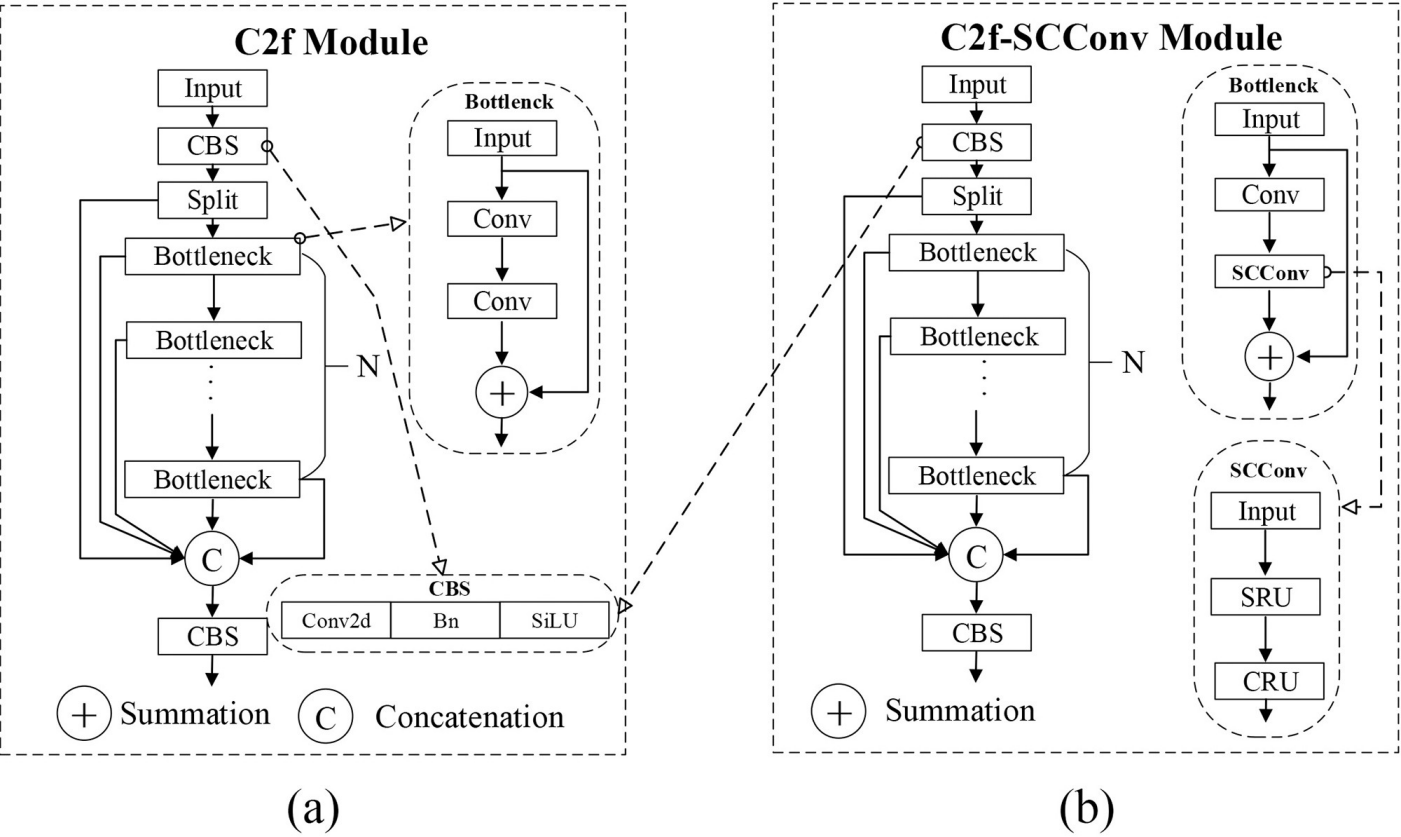
Structure Diagram of the C2f (a) and Improved Modules (b).

This paper proposes the C2f-SCconv module by incorporating the SCconv module into the lightweight improvement of the C2f module in YOLOv8. The structure of the C2f module is shown in Fig 6(a), where the Bottleneck structure in the C2f module performs two CBS convolutions on the input and then adds it to the original input before outputting. The proposed module replaces the second CBS operation in the Bottleneck of the C2f module with the SCconv module. Subsequently, all C2f modules in the Backbone and Neck structures are replaced with C2f-SCconv modules. Fig 6(b) shows the structure of the C2f-SCconv module.

### 3.3 Attention mechanism: Triplet attention module

**Fig 7:**
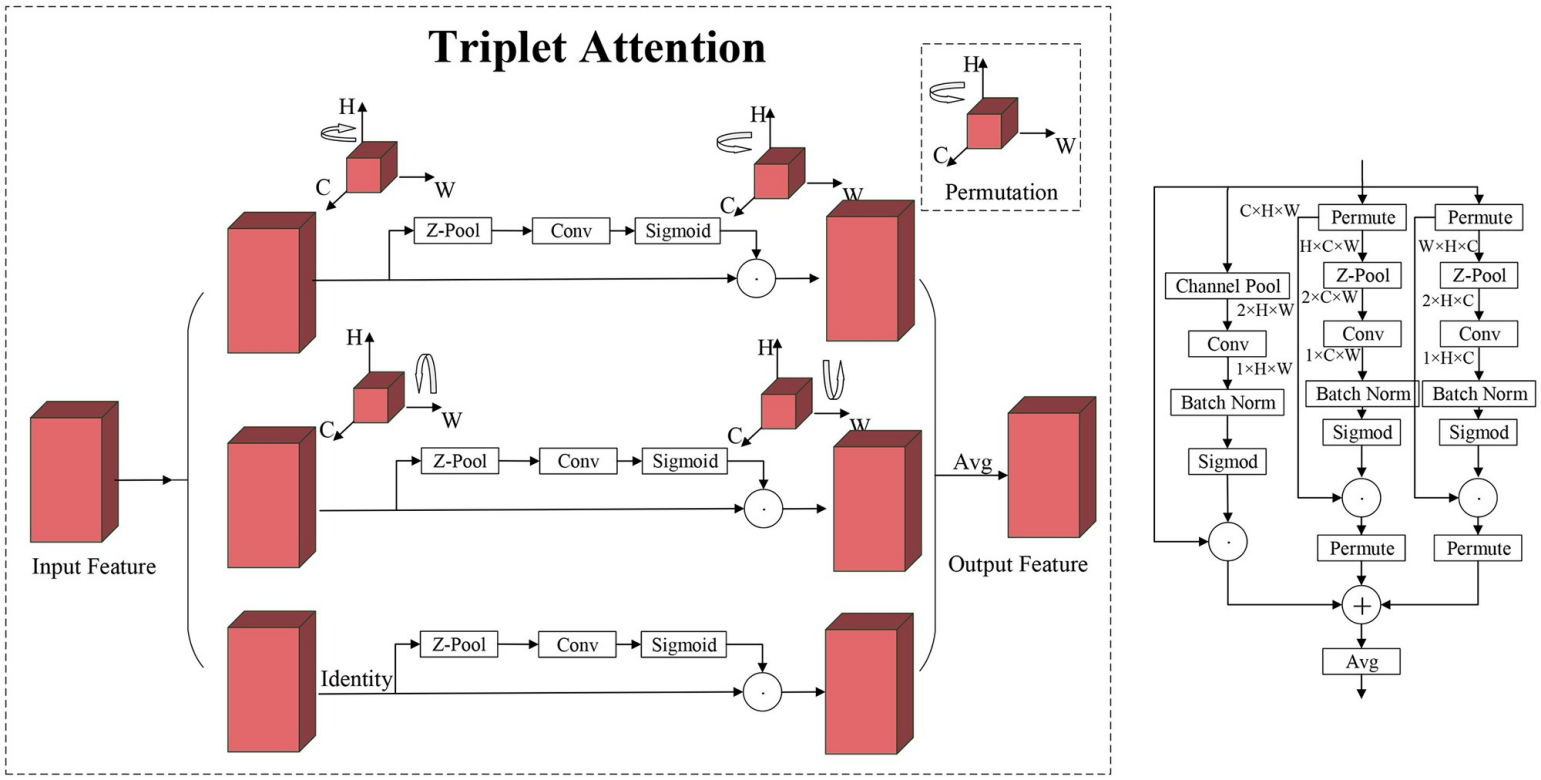
Triplet attention mechanism structure diagram.

In the field of deep learning, attention mechanisms have become a key technology for optimizing neural networks due to their excellent performance and flexibility, significantly improving the efficiency and accuracy of models in handling complex data. With the continuous advancement of artificial intelligence technology, attention mechanisms have become an important research direction for enhancing the learning ability of neural networks. Based on this, we introduced the Triplet Attention [28] mechanism into the Backbone architecture of ST-YOLO to further enhance the model’s feature extraction capability and ensure comprehensive capture of both global and local information in the image. Triple Attention achieves multi-dimensional fine-grained attention to feature maps through the design of spatial, channel, and pixel-level attention mechanisms, thereby significantly improving the efficiency and accuracy of feature extraction. Fig 7 shows the model structure and network structure of Triple Attention.

Triplet Attention includes three branches, which are used to capture the interaction information between the channel dimension (C) and spatial dimensions (W/H), as well as the traditional spatial attention weight calculation. The first branch processes the input features through a Z-Pool layer, then generates spatial attention weights via a 7x7 convolution and a Sigmoid activation function. The second branch transforms the input feature dimensions to H × C × W, performs Z-Pool processing on the H dimension, then converts back to C × H × W after similar operations for element-wise addition. The third branch transforms the input features to W × H × C, performs Z-Pool processing on the W dimension, and follows similar operations to the first two branches, finally converting back to C × H × W. The Z-Pool layer reduces the channel dimension tensor to two dimensions by concatenating average pooling and max pooling features, retaining rich representations while reducing depth and computational load. Finally, the output features of the three branches are summed and averaged to generate the final feature representation, effectively enhancing the performance of the attention mechanism. The calculation of the Z-Pool layer can be expressed by Eq 1.

$$\begin{array}{c} \left[Z-pool(\chi )=[Maxpool_{0d}(X),Avgpool_{0d}(X)]\right] \end{array}$$ (1)

### 3.4 Experimental environment

The operating system for this experiment is Windows 10, the central processor is Intel(R) Core(TM) i5-12400 @ 2.50GHz with 12 cores, and the graphics processor is Nvidia RTX 3060 with 12GB of memory. The experimental code is based on Pytorch. The experimental environment uses Python version 3.8, Pytorch version 1.11.0, and CUDA version 11.3.

### 3.5 Hyperparameter settings

In this experiment, the training epochs are set to 500, the batch size is 16, the initial learning rate is 0.01, the momentum is 0.937, and the weight decay coefficient is 0.0005. The input image size is 640 × 640, and the number of workers is 8. YOLOv8’s mosaic processing is used for data augmentation.

### 3.6 Evaluation metrics

Evaluation metrics are important tools for assessing model quality. The evaluation metrics for model speed used in this paper include computational load (GFLOPs) and the number of parameters. The evaluation metrics for model accuracy include precision (P), average precision for each class (AP), mean average precision across all classes (mAP), and recall rate (R).

Precision is used to measure how many of the samples predicted as positive by the classification model are truly positive examples. The calculation formula is shown in Eq 2.

$$\begin{array}{c} P=\frac{TP}{TP+FP} \end{array}$$ (2)

In the formula, P represents precision, TP stands for the number of true positive samples predicted as positive samples, and FP represents the number of false positive samples predicted as positive samples. Recall rate (R) represents the proportion of defect regions that are correctly predicted as defect regions out of all actual defect regions. The calculation formula is shown in Eq 3:

$$\begin{array}{c} R=\frac{TP}{TP+FN} \end{array}$$ (3)

In the formula, TP and FP are the same as in Eq 2, and FN represents the number of false negative samples predicted as negative samples.

Average Precision (AP) is a commonly used evaluation metric to measure the accuracy of a model in information retrieval or object detection tasks across different categories or thresholds. It evaluates the model’s performance by calculating the area under the Precision-Recall curve. The calculation formula is shown in Eq 4:

$$\begin{array}{c} AP=\int PR\;dR \end{array}$$ (4)

Mean Average Precision (mAP) is used to measure the accuracy of a model across all categories or thresholds in information retrieval or object detection tasks. The calculation formula is shown in Eq 5:

$$\begin{array}{c} mAP=\frac{1}{N}\sum_{i=1}^{N}AP_{i} \end{array}$$ (5)

In the formula, *AP*_*i*_ represents the Average Precision of the i-th class, and N represents the total number of classes. To determine the localization capability of the model, the Intersection over Union (IoU) evaluation metric is introduced. IoU is used to measure the degree of overlap between the predicted bounding box and the ground truth bounding box. The larger the IoU, the closer the predicted position is to the actual position, and the better the effect. The calculation formula is shown in Eq 6:

$$\begin{array}{c} IOU=\frac{\left|A\cap B\right|}{\left|A\cup B\right|} \end{array}$$ (6)

In the formula, A represents the area of the ground truth bounding box, and B represents the area of the predicted bounding box.

To more intuitively display the training effect of the model, the mAP@0.5 comprehensive evaluation metric is introduced. mAP@0.5 represents the mean Average Precision when the IoU value is set to 0.5. When IoU > 0.5, it is considered that there is a predicted target within the predicted bounding box. When IoU < 0.5, it is considered that there is no predicted target within the predicted bounding box. mAP@0.5 can comprehensively evaluate the localization accuracy and classification accuracy of the model. The formula is shown in Eq 7:

$$\begin{array}{c} \text{mAP@0.5}=\frac{1}{N}\;\;\;\;\;\sum_{\begin{array}{c} i=1\\ \text{IOU}=0.5 \end{array}}^{N}\;\;\;\;\;AP_{i} \end{array}$$ (7)

## 4 Results

### 4.1 Comparative experiments

Under the experimental environment provided in this paper, we compared the performance of ST-YOLO with current mainstream object detection algorithms on the PV array infrared defect image validation set. The experimental results are shown in Table 1.

**Table 1:** Model validation results.

Model	The Precision of each category			The Various indicators of whole model				
	DB(%)	TB(%)	DL(%)	P(%)	R(%)	mAP@0.5(%)	FLOPs(G)	Params(M)
SSD300	94.7	91.2	87.2	92.9	90.3	90.6	126.2	145.56
Faster R-CNN	92.2	89.2	83.9	92.4	89.1	88.1	134.1	108.5
YOLOv3	94.4	90.2	86.5	92.8	88	90.1	122.5	120.7
YOLOv4	91.4	88.2	82.6	92.1	86.4	86.7	78.5	244.6
YOLOv6s	94	93.2	85.6	92.5	88.2	89.4	18.8	40.6
YOLOX_s	96.3	92.1	90.9	93.4	90.6	93.4	20.1	16.3
YOLOv5s	95.7	93	91.2	93.3	91.8	94.8	23.8	9.11
YOLOv7-Tiny	89	92.9	**1**	93.9	89.3	93.3	**13**	**6.01**
YOLOv8s	**96.5**	93.1	91.6	93.7	**94.3**	95.2	28.4	11.12
**ST-YOLO**	**96.5**	**93.3**	**1**	**96.6**	93.2	**96.6**	23.6	9.45

From Table 1, it can be concluded that ST-YOLO performs exceptionally well across all metrics, particularly achieving optimal values in accuracy and mAP@0.5. Specifically, ST-YOLO achieves a mAP@0.5 of 96.6%, which is 1.4% higher than the baseline YOLOv8s and 1.8% higher than YOLOv5s, making it the best performer among all compared models. Additionally, ST-YOLO’s overall Precision also reaches 96.6%, demonstrating its advantages in combined precision and recall performance. In terms of parameter count and computational complexity, compared to the baseline YOLOv8s, ST-YOLO reduces model parameters by approximately 15% and GFLOPS by about 17%. Although ST-YOLO has slightly higher parameter counts and computational complexity compared to YOLOv7-Tiny, its overall performance improves significantly. Compared to YOLOv5s, ST-YOLO maintains low parameter counts and computational complexity while significantly enhancing performance. Comprehensive analysis of the performance indicators of various object detection algorithms shows that ST-YOLO achieves the best detection performance while maintaining low parameter counts and computational complexity, demonstrating its overall advantages in performance and efficiency.

**Fig 8:**
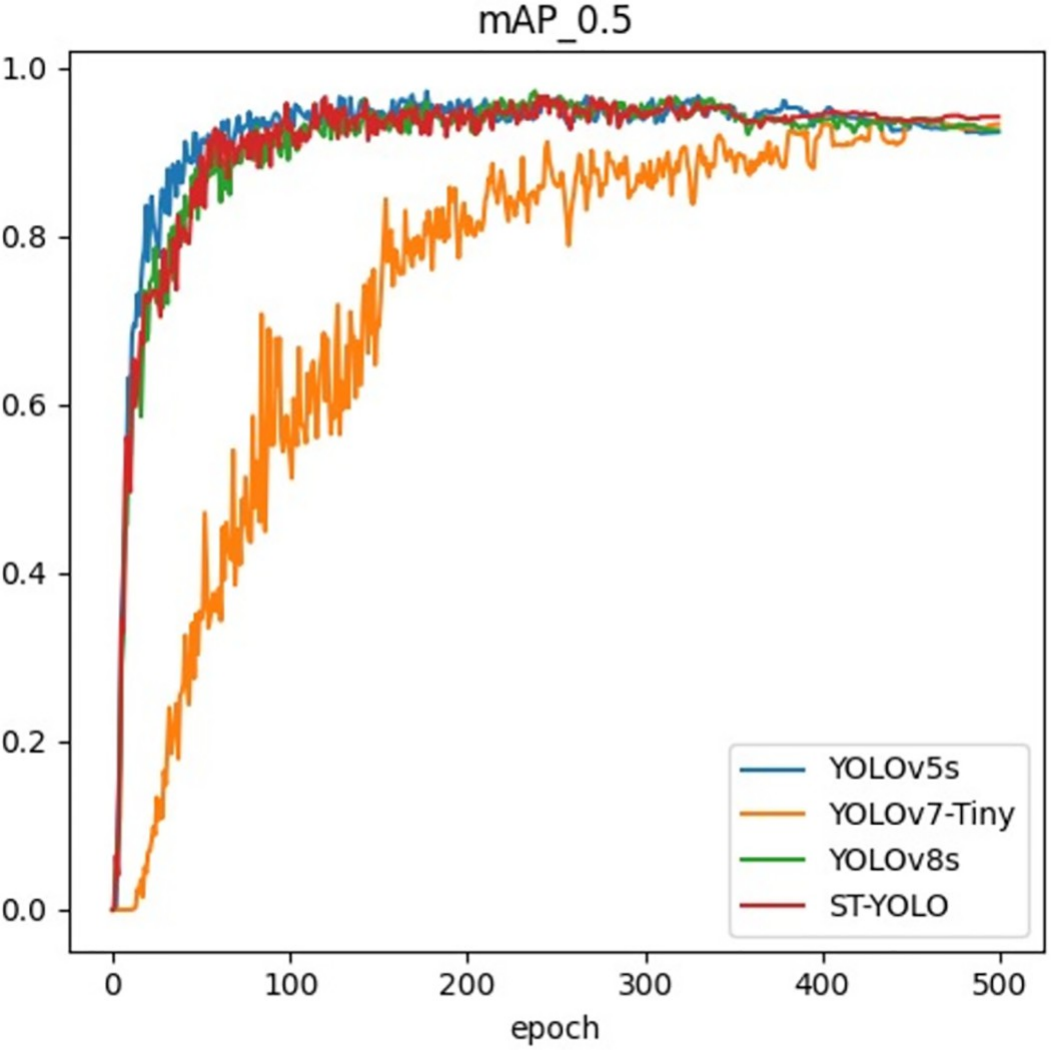
Training curves of mAP@0.5 for four optimal models.

Fig 8 shows the training curves of the four optimal models in Table 1 under the mAP@0.5 metric. It can be seen that the YOLOv5s, YOLOv8s, and ST-YOLO models have a faster convergence rate in the early stages of training and exhibit stable performance during training, gradually approaching convergence. In contrast, the training curve of YOLOv7-Tiny is less stable, with larger fluctuations. Considering the parameter count of each model, ST-YOLO has fewer parameters and lower computational complexity, indicating that ST-YOLO can efficiently utilize computational resources while maintaining high performance. In summary, ST-YOLO not only leads in performance metrics but also excels in training efficiency and stability, making it suitable for deployment in resource-constrained environments and meeting practical application needs.

**Fig 9:**
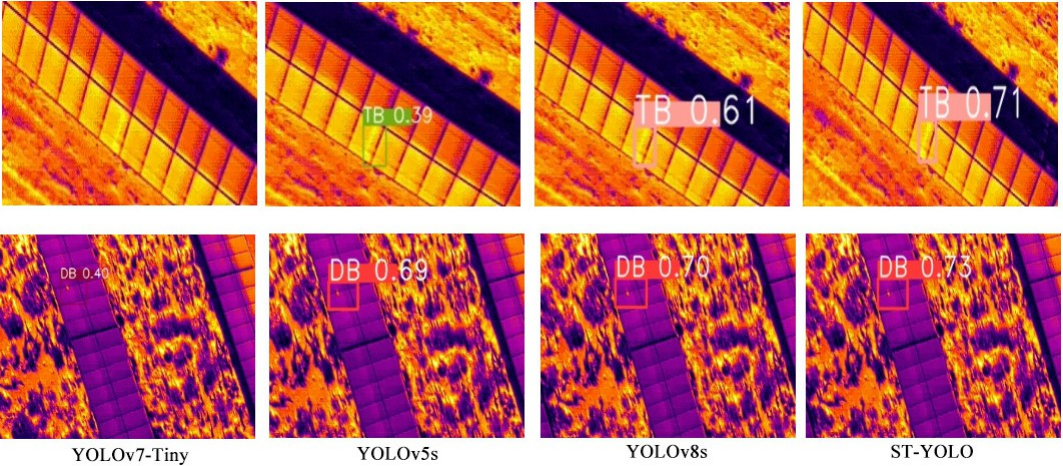
Detection results diagram.

To intuitively verify the effectiveness of ST-YOLO, this paper conducted a visual analysis of the detection results of the four optimal models in Table 1. The detection results of the models are shown in Fig 9. As can be seen from the Fig, only YOLOv7-Tiny exhibited missed detections among the four models. Among all the detection results, ST-YOLO achieved the highest confidence.

### 4.2 Ablation experiments

To validate the effectiveness of the proposed improvements, we conducted ablation experiments on the photovoltaic array infrared defect image dataset. We used mAP@50 and model size as evaluation metrics. The specific experimental results are shown in Table 2. Method A represents the baseline YOLOv8s, with a mAP@0.5 of 95.2% and a model size of 11.12M. Method B introduces the SCconv convolution module to YOLOv8s, achieving a 17.8% reduction in model size. Method C adds the Triplet Attention mechanism to Method A, increasing the model size by 0.27M but improving mAP@0.5 by 0.9%. Method D combines both Method A and Method B, forming the proposed ST-YOLO. The final experiments show that compared to the baseline YOLOv8s, ST-YOLO achieves a 1.4% improvement in mAP@0.5 while reducing the model size by 15%.

**Table 2:** Ablation experiment verification results.

Method	SCconv	Triplet Attention	mAP@0.5	Params(M)
A	-	-	95.2	11.12
B	✓	-	94.5	**9.14**
C	-	✓	96.1	11.39
D	✓	✓	**96.6**	9.45

**Fig 10:**
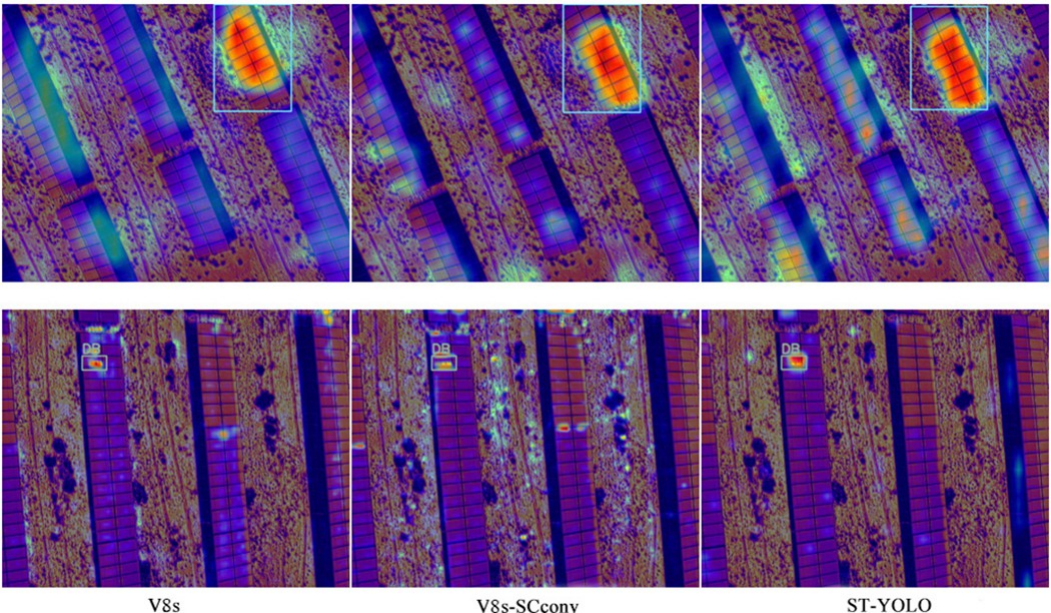
Heatmap comparison diagram.

To more intuitively demonstrate the actual effects of different improvement strategies, we used CAM technology to extract and visualize the feature heatmaps of each strategy. By comparing the heatmaps in Fig 10, we can clearly observe that ST-YOLO shows a significant improvement in detection performance compared to the baseline YOLOv8s. This notable performance fully validates the effectiveness and practicality of the improvement strategies we adopted.

## 5 Conclusions

This paper proposes a method specifically designed for photovoltaic module defect detection called ST-YOLO. This method incorporates the SCconv convolution module and the Triplet Attention mechanism to ensure efficient computation while achieving accurate detection of photovoltaic module defects. First, based on SCconv convolution, we proposed the C2f-SCconv module, which significantly reduces model parameters and enhances detection speed. Second, by integrating the Triplet Attention mechanism into the Backbone, the model’s feature extraction capability is improved without substantially increasing model parameters. Finally, experiments on the self-built photovoltaic array infrared defect image dataset show that compared to the baseline YOLOv8s, ST-YOLO reduces model parameters by about 15%, GFLOPS by about 17%, increases overall accuracy by 2.9%, and improves mAP@0.5 by 1.4%. Analysis and comparison of the model’s feature heatmaps reveal that ST-YOLO is significantly better than the baseline YOLOv8s in feature extraction. In future work, we plan to explore further possibilities of model lightweighting through techniques such as pruning and knowledge distillation to optimize model performance, enhancing operational efficiency while maintaining high accuracy. This will lay a solid foundation for research and application in photovoltaic module defect detection and support the sustainable development of the photovoltaic industry.
